# Heat Exposure, Heat-Related Symptoms and Coping Strategies among Elderly Residents of Urban Slums and Rural Vilages in West Bengal, India

**DOI:** 10.3390/ijerph191912446

**Published:** 2022-09-29

**Authors:** Barun Mukhopadhyay, Charles A. Weitz

**Affiliations:** 1 Biological Anthropology Unit, Indian Statistical Institute, Kolkata 700 108, India; 2 Indian Anthropological Society, Kolkata 700 019, India; 3 Department of Anthropology, Temple University, Philadelphia, PA 19122, USA

**Keywords:** individually experienced heat stress, heat index, gender, comfort prediction

## Abstract

The impact of heat stress among the elderly in India—particularly the elderly poor—has received little or no attention. Consequently, their susceptibility to heat-related illnesses is virtually unknown, as are the strategies they use to avoid, or deal with, the heat. This study examined perceptions of comfort, heat-related symptoms, and coping behaviors of 130 elderly residents of Kolkata slums and 180 elderly residents of rural villages south of Kolkata during a 90-day period when the average 24-h heat indexes were between 38.6 °C and 41.8 °C. Elderly participants in this study reported being comfortable under relatively warm conditions—probably explained by acclimatization to the high level of experienced heat stress. The prevalence of most heat-related symptoms was significantly greater among elderly women, who also were more likely to report multiple symptoms and more severe symptoms. Elderly women in the rural villages were exposed to significantly hotter conditions during the day than elderly men, making it likely that gender differences in symptom frequency, number and severity were related to gender differences in heat stress. Elderly men and elderly village residents made use of a greater array of heat-coping behaviors and exhibited fewer heat-related symptoms than elderly women and elderly slum residents. Overall, heat measurements and heat-related symptoms were less likely to be significant predictors of most coping strategies than personal characteristics, building structures and location. This suggests that heat-coping behaviors during hot weather were the result of complex, culturally influenced decisions based on many different considerations besides just heat stress.

## 1. Introduction

Climate change is exposing a growing proportion of the world’s population to extreme heat conditions. These conditions are particularly stressful for the elderly, since several irreversible physiological changes occur with age, making it more difficult for them to maintain thermal equilibrium in hot environments [1,2]. These include a reduction in cardiac functioning, as well as changes in the structure and function of peripheral vessels, resulting in a significant reduction in blood flow from the body core to the skin, less cutaneous vasodilation, and a delay in the onset of vasodilation [3,4]. Additionally, the initiation of sweating among the elderly occurs at a slightly higher skin temperature than for younger people [5], while sweat volume and sweat duration are reduced [6]. Consequently, heat transfer from the body is reduced, and core temperatures are elevated [7,8,9,10].

Because these changes reduce heat tolerance, the elderly are commonly characterized as experiencing elevated risk for both heat-related mortality and heat-related illnesses [11,12,13,14,15,16,17], particularly if they have pre-existing medical concerns [12,18,19,20]. These characterizations tend to be based on country-wide or regionally aggregated information on total deaths (by age) during hot weather and on regional or country-wide hospital admissions during hot weather [21,22,23]. Depending on available data, these studies can provide information about general risk factors (predominately demographic; but also information on cause of death, and on comorbidities) [18,19]. However, these studies rarely include fine-grained information on individual attitudes, behaviors and experienced conditions that might be used to understand the health consequences of heat stress among the elderly in different cultural contexts.

This information is provided by a growing number of smaller-scale, community-based studies that report on the thermal comfort of elderly individuals during hot weather [24,25,26], about heat perceptions/attitudes of the elderly [27,28,29,30], about the coping strategies used by the elderly during hot weather [25,26,31,32,33,34], and about heat-related symptoms among the elderly [35,36]. Some studies also have included information about indoor heat stress in dwellings inhabited by the elderly [37,38], based on the understanding that external conditions may not represent the degree of heat stress experienced by the elderly who tend to remain indoors during hot weather [19,34,39].

Most studies describing the responses of elderly individuals during hot weather have taken place in temperate environments—either during summer periods when outdoor temperatures occasionally exceed 30 °C [37,39,40], or during heat waves when outdoor temperatures exceed 35–40 °C for several days, e.g., [36,41] or as post hoc assessments after hot weather periods, e.g., [26,27]. Comparable studies of the elderly have rarely taken pace in countries located in the tropics, such as India, where the intensity of summer heat is often much greater and lasts longer than in temperate climates [42], where heat waves are more intense [43], and where both are becoming more severe [44,45,46].

Studies linking thermal conditions experienced by the elderly in India to heat-related symptoms, illnesses or coping strategies are practically non-existent. Two community studies [47,48] indicate that elderly Indians are at greater risk for “heat-related symptoms” or “heat-related illness”, but provide no specifics. Aside from a report that elderly in rural villages may spend more time indoors during the hottest times of the day [47], Indian studies that include information on heat coping strategies [49,50,51,52,53] offer no information about specific behaviors used by the aged. Finally, the few Indian heat studies that have included elderly participants ignore individual variability in heat exposure due to differences in activity and time spent in indoor and outdoor conditions. Instead, the severity of experienced heat is characterized by measurements made at stationary indoor or outdoor facilities—a common strategy in Indian heat studies [53,54,55,56,57,58]—but one that can misclassify heat stress [59,60]. A more accurate assessment of heat exposure can be achieved by using small telemetric devices that can be unobtrusively worn throughout the day and night to continuously record environmental conditions regardless of location [59,60,61].

Thus, this study uses measurements of individually experienced temperatures, humidity and heat index to understand how differences in the degree of heat stress experienced by a large sample of elderly Indians affect their perception of comfort, the type and prevalence of heat-related symptoms, and the coping strategies they used to manage hot conditions. Three specific aspects of this general goal are explored: (1) a comparison between elderly living in an urban slum and elderly living in rural villages; (2) a comparison between elderly women and elderly men; and (3) a determination of the extent to which different coping behaviors are likely to be related to experienced heat, to heat-related symptoms or to demographic/behavioral characteristics. This approach, which has been used to understand how experienced heat conditions operate to structure health risk and resilience in other studies [60,62,63], will provide information that, for the first time, focuses on the health of elderly Indians living in hot conditions.

## 2. Materials and Methods

### 2.1. Study Location

This research was conducted at two locations in the eastern Indian state of West Bengal. The first study site consisted of registered slums, also known as *Bustees* [64], located in two wards in north central Kolkata, a city that has been identified as having a particularly high level of heat vulnerability [65]. The study area was characterized by densely compacted, single-room, low-rise dwellings, constructed mostly of cement walls and corrugated iron or tile roofs [66]. Research also was simultaneously conducted in a cluster of rural villages, located about 75 km south of Kolkata. The villages were situated in a rice-growing region of West Bengal and thus were representative of rural agricultural areas of West Bengal that experience significant thermal stress during hot summers [67,68]. There, dwellings were separated from each other, and most were surrounded by gardens, fields or other naturally vegetated areas, as well as by water ponds.

### 2.2. Study Participants

The study took place during a 90-day period between the end of May and the end of August 2019, coincidental with the hottest summer months in the region. In the two municipal wards of Kolkata where the slums were located, electoral roll lists were used to identify potential elderly participants. Our sampling strategy consisted of visiting all dwellings occupied by an elderly resident (60 years and older), at which time the study procedure was described verbally to potential participants and provided in writing. Our goal was to contact and enroll as many elderly participants as possible. The only requirement for enrollment was age equal to, or greater than, 60 years. After age verification (usually some form of identification made during the household visits), a total of 153 elderly residents of 123 households were interviewed, and 130 agreed to participate in the personal monitoring study. In the rural villages, potential participants were identified based on lists of elderly residents maintained by a local hospital. As in Kolkata, we contacted as many potential study participants as time and equipment limitations would permit. After age verification, a total of 253 elderly residents of 101 rural village households were interviewed, and 180 agreed to participate in the personal monitoring study.

### 2.3. Data Collection

**Figure 1:**
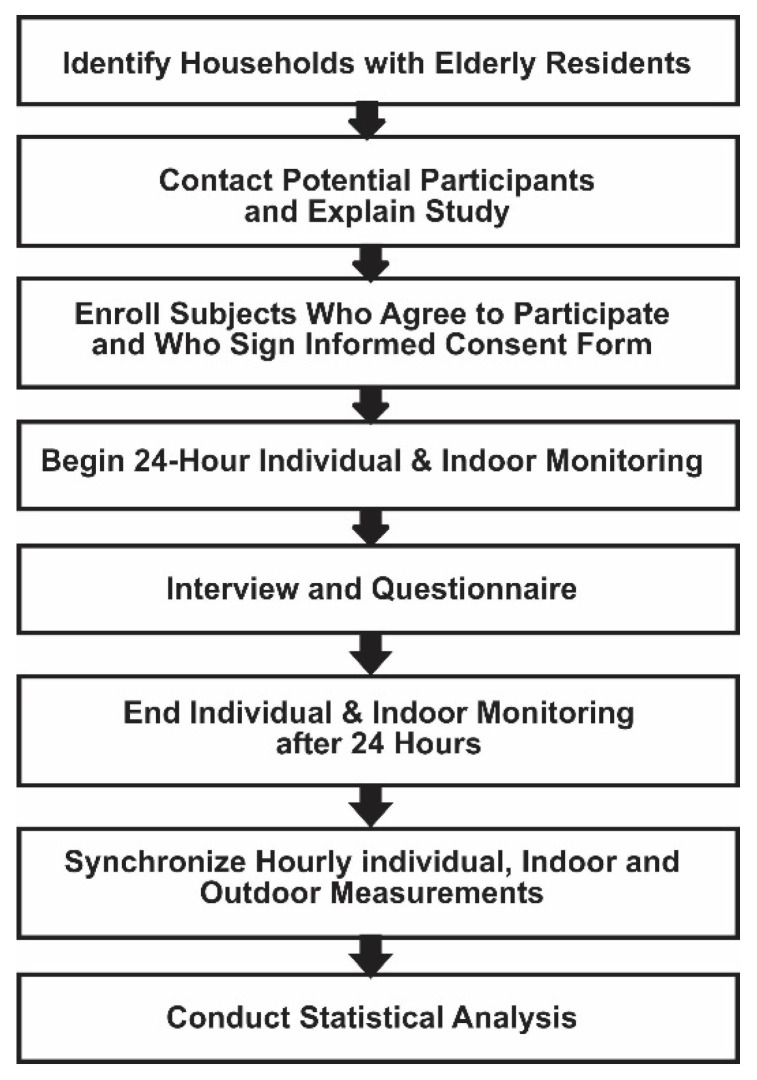
Flow chart of data collection process.

Figure 1 shows a flow chart of the basic steps followed during the study. Once a participant was enrolled in the study, the temperatures, humidity and heat indexes they experienced over a 24-h period were continuously monitored using a Kestrel Drop D2^®^ monitor (Kestrel Meters, Boothwyn, PA 19061 USA) (Instrument specifications are available in Supplemental File S1). The Kestrel D2 individual monitors were attached to a lanyard that was suspended around the neck of participants so that it could be worn outside clothing. Individuals were instructed to wear the device continually–although when asleep, they were told that they could position the monitor near where they slept, but to put it on again if they awoke during the night. During the same 24-h period, interior temperature, humidity and heat index of dwellings were monitored using a Kestrel 5400 Heat Stress Tracker^®^ (Kestrel Meters, Boothwyn, PA 19061 USA) (Instrument specifications are available in Supplemental File S1). The Kestrel 5400 room monitors were placed on a wall or shelf approximately 1.5 m above the floor and away from direct heat sources, such as cookstoves see [69]. In both the urban slum and the rural village, a trained member of the research team was present to install and remove the Kestrel Drop D2 and Kestrel 5400 Heat Stress Tracker monitoring devices, give instructions to the participants, and answer questions. At the end of each 24-h period, the data were downloaded, and the devices were recalibrated before they were used again.

A 24-h monitoring period had several advantages. First, it permitted the study to include a large number of participants over a wide range of heat conditions during the 3-month study period. As noted in other public health studies, e.g., [70], this cross-sectional strategy provides an optimal way of understanding the effects of temporal variation. Second, the larger sample size produced by this strategy made possible a clear determination of statistical significance using a variety of statistical analyses (see below). Third, a shorter monitoring period resulted in compliance with the monitoring protocol. This was apparent during a pre-study and continued to be the case during the main study.

Outdoor temperature and humidity measurements were recorded hourly at Indian Meteorological Department (Government of India) weather stations in Kolkata (approximately 3 km from the urban study site) and Diamond Harbor (approximately 25 km from the rural study site). The outdoor heat index (HI) was computed using a formula developed for the United States National Weather Service [71] (see Supplemental File S2), which closely approximates Steadman’s original tables for subjective heat assessment under different heat and humidity conditions [72]. The HI recorded by both the Kestrel Drop D2 and the Kestrel 5400 Heat Stress Monitor was determined by an internal algorithm using the same formula.

For purposes of analysis, the timing of indoor measurements made by the Kestrel 5400 Heat Stress Tracker and the personal measurements made by the Kestrel Drop D2 were synchronized with the hourly outdoor measurements. Thus, at each hour during the 24-h monitoring period (i.e., at 12 A.M., 1 A.M., 2 A.M., 3 A.M., and so on), the individually experienced temperatures, humidity and heat indexes of participants corresponded to temperatures, humidity and heat index measurements made indoors and outdoors.

During the same day that participants were monitored, usually during the afternoon, a questionnaire (Supplemental File S3) was administered in Bengali as part of the interview process. The questionnaire included basic demographic information (age, gender, marital status, education, whether an individual continued to engage in salaried work or was retired, and household composition), along with information about activity pattern, smoking status, and existing health problems. Additionally, dwelling characteristics were noted (construction materials, number of rooms, existence and location of windows). Individuals were asked if they were uncomfortable or comfortable in the heat during the 24-h monitoring period, and if they experienced any or all of the following heat-related symptoms: disturbed sleep, excessive sweating, excessive thirst, muscle cramps, fatigue/weakness, dizziness, headache, nausea or vomiting, fainting, and prickly heat/heat rashes. While these are not all the potential heat-related symptoms, they are among the most commonly reported [73,74]. Individuals also were asked whether they practiced any or all of the following heat coping strategies: resting, drinking water, moving to a cooler location, using an electric fan in sleeping area, using a hand fan, reducing or omitting household/economic activities, altering, reducing or omitting social activities, changing or removing clothing, taking a shower, bath or sponge bath, adding food considered to be culturally appropriate for consumption during the summer, and deleting foods considered to be culturally inappropriate for consumption during the summer. 

To ensure that all interviews were conducted in a manner that was as similar as possible, day-long training sessions were held for the individuals who administered the questionnaires. Mock interviews were conducted iteratively, and the performance of each questionnaire administrator was evaluated and refined. In practice, most interviews took between 30 and 45 min to conduct.

All questions on thermal comfort, heat-related symptoms and heat-related coping strategies were asked in a binary answer format (yes/no). There were two reasons for this. First, based on a plot study, we determined that binary response questions were easier to communicate, easier for potential respondents to understand, and easier for them to answer. Second, research has indicated that information obtained from binary response questions leads to results that are equally reliable as those obtained by ordinal multi-category questions [75], can be used effectively to gauge subjective assessments [76], and can be used in health surveys with equal validity as multi-category answer questions [77]. Questions on thermal sensation were asked both positively and negatively (at different points in the interview) to identify potential acquiescence bias.

### 2.4. Statistical Analysis

Differences between the rural villages and Kolkata slums in the effects of heat stress and between men and women were assessed in two ways: first, by comparing the frequency of responses to the binary questions about comfort, heat-related symptoms and coping behaviors, using a chi-square analysis; and second, by using odds ratios to determine the extent to which location, gender and a set of other demographic and behavioral characteristics (age group, marital status, employment, smoking, and activity) were related to the likelihood of reporting heat-related symptoms and heat-related coping strategies.

Binary logistic regressions were used to evaluate the relationship between individual experienced heat conditions and perceptions of comfort and to identify significant conditions that affected the likelihood of reporting each of the heat-coping strategies. The binary logistic regression used to determine the odds of reporting comfort vs. discomfort relative to median individual experienced heat index (IEHI) = exp [b_o_ + b_1_(IEHI)]. where exp = exponential function (2.72); b_o_ =constant or intercept of the regression equation; b_1_ = the coefficient associated with the predictor variable (IEHI).

The probability that a specific median individually experienced heat index (IEHI) value will be associated with reporting comfort vs. discomfort =

$$\frac{\mathrm{odds}\;\mathrm{associated}\;\mathrm{with}\;a\;\mathrm{specific}\;\mathrm{IEHI}\;\mathrm{value}}{1+\;\mathrm{odds}\;\mathrm{associated}\;\mathrm{with}\;a\;\mathrm{specific}\;\mathrm{IEHI}\;\mathrm{value}}$$

$$\frac{\mathrm{bo}\;+\;b1\;\left(\mathrm{specific}\;\mathrm{IEHI}\;\mathrm{value}\right)\;}{1+\left[\mathrm{bo}\;+\;b1\;\left(\mathrm{specific}\;\mathrm{IEHI}\;\mathrm{value}\right)\right]}$$

This equation can be iterated at different IEHI values to determine the median IEHI value at which 80% and 90% of individuals report being comfortable.

To determine the odds of reporting a specific coping strategy relative to IEHI, heat-related symptoms, demographic and residential characteristics, a binary logistic regression with multiple predictors is used:

Odds of reporting yes/no for a coping strategy = exp [b_o_ + b_1_ (PCA of 24-h IEHI) + b_2_ (symptoms) + b_3_ (age) + b_4_ (gender) + b_5_ (location) + b_6_ (marital status) + b_7_ (employment) + b_8_ (tobacco use) + b_9_ (education) + b_10_ (activity) + b_11_ (number of rooms in dwelling) + b_12_ (number of co-inhabitants) + b_13_ (dwelling wall material) + b_14_ (dwelling roof material)]

Details of the model are presented in Supplemental File S4.

The sample size of 310 in this study is well within suggested numbers for both principal component analysis [78] and binary logistic regression analysis that include the number of independent variables noted above [79]. All analyses were preformed using IBM SPSS Statistics for Windows, Version 26.0. (IBM Corp.: Armonk, NY, USA).

## 3. Results

### 3.1. Participant Characteristics

The proportion of elderly women was greater in the Kolkata slums than in the rural villages, and so was the proportion of un-married individuals (single, divorced widowed/widowers). However, there was a greater proportion of active participants (particularly those who were active all day) in the rural villages (see Table 1). There were no location differences in the proportion of older versus younger elderly, in the proportions of individuals with different education backgrounds, in the proportions of employed vs. unemployed, or in the proportions of individuals who used/did not use tobacco.

### 3.2. Heat Conditions during the Study

A summary of the outdoor, indoor and individually experienced temperature, humidity and heat indexes (Table 2) shows that indoor HI measurements in the Kolkata slums were hotter than outdoor HI measurements throughout the day and particularly at night. In the rural villages, indoor HI measurements were somewhat cooler than outdoor HI measurements during the day, and then declined to outdoor levels during the overnight hours. Elderly women and men in the Kolkata slums experienced similar heat levels during the daytime and at night. However, in the rural villages, elderly women experienced higher HI levels than elderly men during the day.

### 3.3. Discomfort/Comfort in the Heat

A determination of the individually experienced 24-h median temperatures and HI at which there was an 80% probability of reporting comfort and a 90% probability of reporting comfort (Table 3a,b) indicates that elderly Kolkata slum residents tolerated warmer conditions than elderly residents of the rural villages. In addition, the high median experienced HI values (which include humidity) suggest a much greater heat tolerance than indicated by temperature alone.

### 3.4. Heat-Related Symptoms

**Figure 2:**
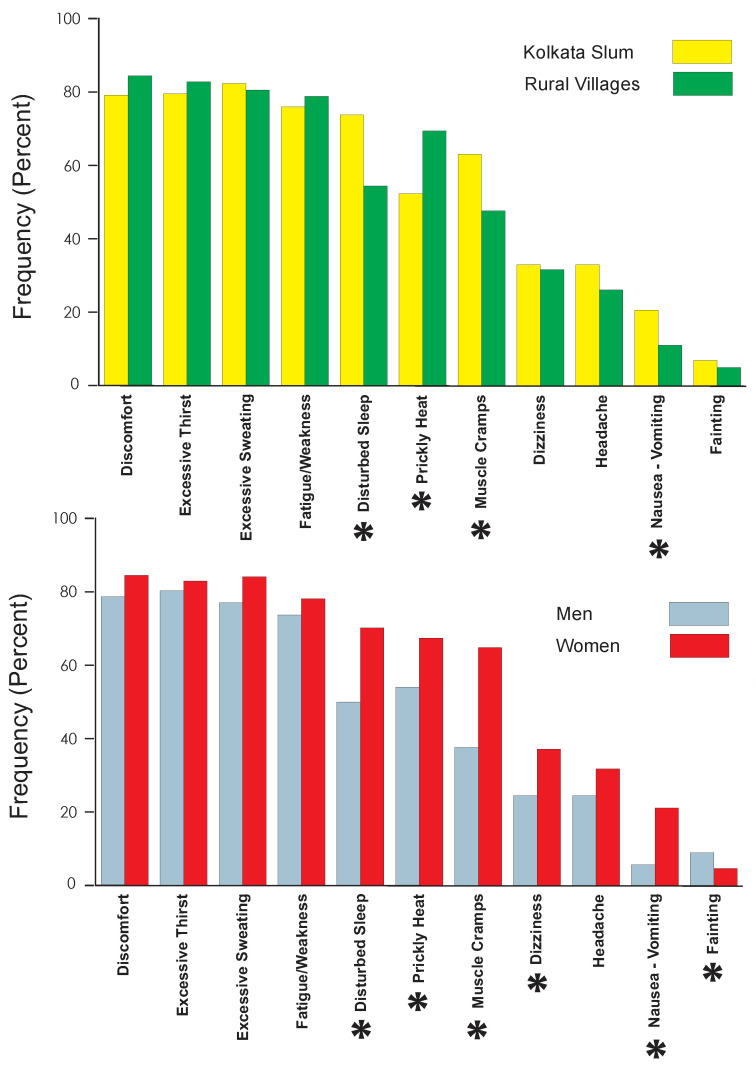
Distribution of symptoms by location (upper panel) and by gender (lower panel). Asterisks indicate statistically significant differences (see Supplemental File S5 for data).

Besides general discomfort, the most commonly reported heat-related symptoms were excessive sweating and fatigue/weakness (Figure 2, Supplemental File S5). There were several important gender differences. Elderly women were significantly more likely to report nausea/vomiting, muscle cramps, disturbed sleep, prickly heat, and dizziness than elderly men (Table 4). Elderly residents of rural villages were significantly less likely than elderly slum residents to report disturbed sleep, nausea/vomiting or muscle cramps; but significantly more likely to report prickly heat.

Further analysis (See Supplemental File S6) indicates that individuals with higher (i.e., post-secondary) education were significantly less likely to report excessive sweating, disturbed sleep, fatigue/weakness, prickly heat, muscle cramps and dizziness compared to individuals with less or no education. No other demographic or behavioral characteristic showed consistently significant odds ratios associated with reporting heat-related symptoms. Individually experienced HI monitored during the 24-h study period provided very little explanatory power concerning heat-related symptoms (Supplemental File S7).

The total number of heat related symptoms reported by residents of the Kolkata slums did not differ significantly from the number reported by residents of the rural village (Kolkata slums = 6.3, Rural Villages = 6.0, F = 1.379, *p* = 0.241). However, elderly women reported significantly more symptoms than elderly men (Women = 6.5, Men = 5.4, F = 20.064, *p* <0.001). The distribution (Supplemental Figure S1) was characterized by a tendency for elderly women to report 7 or more symptoms, while elderly men tended to report 6 or fewer (chi-square = 29.859, *p* < 0.001).

Median 24-h experienced HI levels did not differ among elderly women or men with different numbers of heat-related symptoms, regardless of their location. However, in the rural villages, elderly women who reported 4 to 7 symptoms exhibited significantly higher median IEHI values than elderly men with the same number of symptoms (Table 5). This difference was exacerbated when the number of symptoms was related to experienced HI at 1 P.M., generally the hottest time of the day. At that time of the day, elderly women in both locations who experienced higher HI levels reported significantly more heat-related symptoms. Furthermore, the HI experienced by elderly village women who reported between 8 and 11 heat-related symptoms was significantly hotter than elderly men.

Very few of the study participants (3 in the Kolkata slums and 8 in the rural villages) reported ever having been admitted to hospital for heat-related illnesses. Self-reported co-morbidities showed few significant associations with heat-related symptoms (Supplemental File S8). However, elderly individuals with no co-morbidities had significantly lower odds of reporting excessive sweating, fatigue or weakness, disturbed sleep, dizziness and fainting.

### 3.5. Heat Coping Strategies

**Figure 3:**
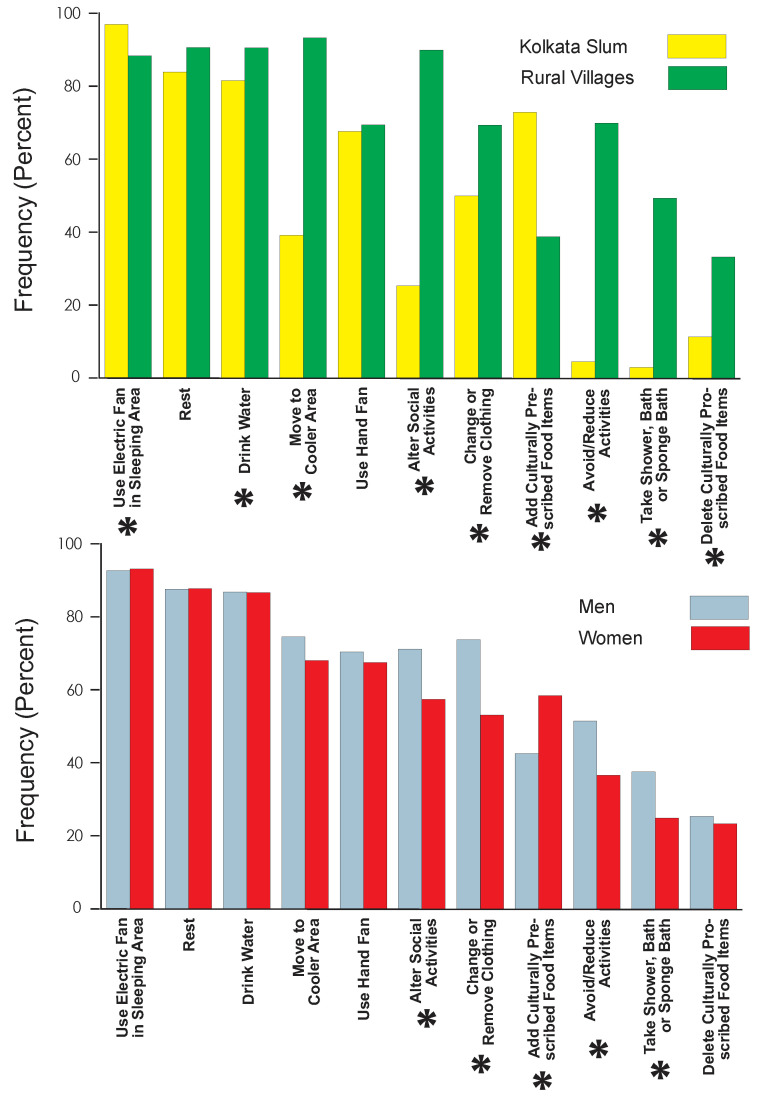
Distribution of heat-coping behaviors by location (upper panel) and by gender (lower panel). Asterisks indicate statistically significant differences (see Supplemental File S5 for data).

There were considerable location differences in the pattern of heat coping strategies (Figure 3). Chi-square analysis (Supplemental File S5) and odds ratio computations (Supplemental File S9) indicate that a significantly greater proportion of elderly village residents reported reducing household/economic activities, altering/reducing social activities, taking a shower or bath, moving to a cooler location, deleting food items considered to be inappropriate for the heat, changing or removing clothing, and increasing water consumption. The only coping strategies significantly less common in the rural villages than in Kolkata were using electric fans in sleeping areas at night and adding food items considered to be appropriate for the hot weather.

Gender differences in coping strategies were less common (Supplemental Files S5 and S9). Elderly women were significantly less likely than elderly men to change or remove clothing, alter or reduce social activities, avoid or reduce household/economic activities, or to take a bath or shower. Elderly women were significantly more likely to add food considered to be appropriate for the hot weather.

A binary logistic regression analysis was performed to determine the extent to which the likelihood of reporting each heat coping strategy was significantly predicted by heat-related symptoms, several participant characteristics (including location and gender), individually experienced HI, and housing characteristics (roofing and wall materials, number of rooms in a dwelling, and number of occupants in a dwelling). Results are summarized in Table 6 (and presented in full in Supplemental File S10). While symptoms were included in the lists of significant predictors of the likelihood of reporting most coping strategies (taking a rest, consuming water, moving to a cooler area, using a hand fan, changing or removing clothing, adding and deleting food items, and avoiding/reducing household/economic activities), participant characteristics and dwelling characteristics were far more common, and generally were more significant. In several cases, the predictors permitted high degrees of sensitivity and specificity. The model that included 4 significant predictors of “move to cooler location” correctly classified 84.5% of the positive responses, and 79.3% of the negative responses; the model that included the 5 significant predictors (which includes no symptoms) of “alter/reduce social activities” correctly classified 86.8% of the positive responses, and 85.2% of the negative responses; the model that included the 5 significant predictors of “take shower or bath” correctly classified 80.6% of the positive responses, and 76.8% of the negative responses; and the model that included the 5 significant predictors of “avoid or reduce household/economic activities” correctly classified 90.8% of the positive responses, and 77.2% of the negative responses In these cases, and in most others, location, housing characteristics and individual characteristics tended to be more significant predictors of the likelihood of reporting a particular coping strategy than symptoms or experienced HI.

## 4. Discussion

During a 90-day study period, elderly residents of Kolkata slums and rural villages in West Bengal were exposed to median temperatures around 30 °C, humidity levels between 70% and 80%, and median heat indexes above 40 °C. Even though elderly residents of both locations reported being comfortable in temperatures that were above those associated with comfort in temperate environments, almost all the participants in this study reported one or more heat-related symptoms. Elderly women were more likely to report most heat-related symptoms, were more likely to report multiple symptoms, and were more likely to report severe symptoms compared to elderly men. In rural villages, this seems associated with significantly greater heat exposure among elderly women. However, even in the Kolkata slums, where gender differences in heat exposure did not exist, elderly women tended to report multiple symptoms. Elderly residents of the rural villages were significantly more likely to use a variety of coping strategies compared to elderly slum residents. The likelihood of using most coping strategies was associated with personal characteristics, building structures and location more than with heat measurements or heat-related symptoms, suggesting that decisions about behaviors during hot weather may be significantly influenced by resource availability (e.g., ponds in the rural villages), economics (e.g., education) and socio-cultural factors, as much as they are by heat or heat-related symptoms.

Logistic regression indicated that the 80% and 90% probabilities of reporting comfort occurred at higher median 24-h individually experienced temperatures and heat indexes among elderly Kolkata slum residents compared to elderly rural villagers. Although this seems to suggest that elderly Kolkata slum residents may exhibit a somewhat greater heat tolerance or acceptance than elderly rural villagers, both groups appear to be comfortable in warm environments—a conclusion that has been reached in a number of other Indian studies, using a variety of different methodologies [50,53,55,56,69,80,81,82].

Despite their apparent tolerance of relatively warm temperatures, the elderly participants in this study—particularly elderly women—reported numerous heat-related symptoms. More elderly women (74.4%) reported 6 or more symptoms than elderly men (48.4%) (chi-square = 20.032, *p* < 0.001). In addition, elderly women reported significantly greater frequencies of disturbed sleep, prickly heat, muscle cramps, and were far more likely to report serious symptoms, such as dizziness and nausea or vomiting Our investigation supports the conclusion that this disparity resulted from differences in heat exposure. During the day in the rural villages (between 7 A.M. and 9 P.M.), the mean individually experienced heat index of elderly women was significantly higher than that of their husbands who were monitored during the same time period [83]. Overall, in the rural village, when adjusting for variation in median 24-h outdoor and indoor heat indexes, elderly women were exposed to dangerous heat levels (i.e., heat indexes ≥ 45 °C) for almost 2 ½ hours longer than elderly men (women = 11.5 h, men = 9.2 h, ANCOVA F = 42.560, *p* < 0.001). To a certain extent, this reflects the fact that more elderly village women reported being active all day compared to elderly village men, while more elderly men reported being inactive/sedentary compared to elderly women [83]. However, even comparing only individuals who were active all day long, elderly village women were exposed to dangerous heat index levels for longer periods than elderly village men (women: *n* = 58, adjusted mean = 11.4 h; men: *n* = 23, adjusted mean = 10.1 h; ANCOVA F = 6.344, *p* = 0.014) and were 4 times more likely to report six or more heat-related illnesses (Odds ratio = 4.083, 95% CI = 1.478, 11.282, Z score = 2.713, *p* value = 0.003). The extremely high degree of heat stress experienced by elderly women in the rural villages—particularly at mid-day—was likely implicated in the larger number of heat-related symptoms they reported—a conclusion supported by other research showing that elderly women who are active outdoors in the heat experience greater heat strain than similarly aged outdoor-active elderly men [84], and by world-wide data indicating that elderly women are more susceptible to heat-related illnesses [85,86] and heat-related death [13,20,87] than elderly men. Elderly women also suffer greater physiological vulnerability to the heat due to a lower capacity to sweat and release heat—possibly related to lower fitness levels—higher body fat percentage, a poorer acclimatization ability [88,89]. However, until studies of physiological adjustments to the heat among elderly living in rural villages and urban slums in India are conducted, it will be impossible to determine the extent to which heat acclimatization exists in different Indian sub-groups, and the potential contribution that acclimatization makes to male-female or rural-urban differences in heat-related symptoms.

It has been suggested that long-term exposure to the heat in India produces a psychological accommodation leading to what has been called the “thermal acceptance” of warmer conditions [50,55,82]. A similar phenomenon has been observed among healthy and socially active elderly in the United States and Europe [26], and also seems to have occurred in our study. Elderly individuals who reported 3 or fewer heat-related symptoms were 6.303 times more likely to report being comfortable in the heat compared to elderly individuals who reported 4–7 heat-related symptoms (95% CI = 3.050, 13.027, z-score = 4.970, *p* < 0.001) and were 34.3 times more likely to report being comfortable in the heat than individuals with 8 or more heat-related symptoms (95% CI = 9.330, 126.087, z-score = 5.322, *p* < 0.001). On the other hand, even though individuals with no chronic diseases also were more likely to report feeling comfortable in the heat than those with chronic diseases (OR = 1.529; 95% CI = 0.789, 2.960), differences were not statistically significant (z-score = 1.258, *p* = 0.104). Further research will be necessary to fully understand psychological perceptions of comfort in the heat.

One thing that appears to have been true among the elderly participants in this study is that comorbidities, which frequently exist, do not often result in hospitalization for heat related illnesses. This is unusual and may represent the fact that elderly Indians may not seek out or may not be referred to physicians who would be likely to order admissions (51).

Adaptive thermal comfort studies in India have tended to focus on the potential benefits of a narrow range of heat-coping behaviors-primarily opening doors/windows, clothing adjustments and the use of fans [47,49,50,56,90,91]. While participants in this study also used these strategies, they were far from universally practiced, and often ineffective. Adjusting clothing was significantly more likely to occur among elderly men and in the rural villages; but considerably less common among elderly women and in the Kolkata slums. Among elderly residents in Kolkata, overnight HI predicted the use of overhead fans, like results from a study of low-income public housing in Mumbai [91]. However, the efficacy of fan use in our study was limited both by the lack of fans in several—particularly rural—households and by intermittent electrical failures. Indoor temperatures in Kolkata slum dwellings were cooler at night if people left their doors open. However, most residents closed their doors and windows at night because of security concerns and to keep out vermin. Furthermore, opening windows was not possible in several Kolkata slum dwellings because they lacked windows [66]. Thus, if adaptive thermal comfort studies are to be extended in the future to cover the vast numbers of Indians living in slums and rural villages, they will have to be modified to include a wider range of heat-coping behaviors.

The array of heat-coping strategies in this study was similar to those reported in studies of rural villages in Maharashtra [47] and urban slums in Ahmedabad [48], even though participants in these studies were younger than elderly village and slum residents included in the present study. This similarity suggests that the coping strategies used by slum dwellers in India may be comparable, regardless of their location or their age; and, likewise, a pattern of similar coping strategies may exist among rural village residents.

Most studies of behavioral adjustments in India seem to link heat-coping strategies directly to the heat [49,50,52,90,91]. In this study, however, we found that the primary predictors of coping strategies tended to not to be HI measured during the 24-h study period, nor heat-related symptoms, nor even reports of discomfort in the heat. There were obvious exceptions, such as excessive thirst being the primary predictor of increased water consumption and higher HI levels in the afternoon being the primary predictor of hand fan use. However, location (Kolkata slums or rural villages), the materials out of which dwellings were constructed, the size and room number of dwellings, and personal or behavioral characteristics (gender, activity, education) provided the greatest number of significant predictors. The large number of non-heat related predictors suggests that practicing any heat coping strategy was the result of complex, culturally influenced decision-making processes based on many different factors besides just heat stress.

The components of this decision-making process, however they are organized, seem to provide elderly rural villagers with a wider variety of heat-coping options compared to elderly slum dwellers. In rural villages, there existed greater opportunities to move to cooler, shaded areas. Verandas were common and open to natural breezes. Trees were relatively abundant and likewise offered respite from direct sunlight, whereas in the Kolkata slums, almost all the elderly who did not work (92 out of 130 total) spent the day watching television indoors in conditions that were significantly hotter than those outdoors. In the rural village, ponds were common and frequently used by men (significantly more than women) to cool off during the day. No such opportunity existed in the slums, and limited water availability and limited privacy meant that showers or sponge baths were not generally used. Elderly men, particularly those who were active in the rural village, also were more likely to change or remove clothing than elderly women. This may have been linked to the fact that elderly men in the rural village were likely to bathe in one of the ponds during the day, or simply because this was a strategy that was available to men and not to women.

Another advantage of life in the rural villages included in this study was the existence of robust social networks [92,93]. Thus, reduction in social activity, while not a preference, was possible in the rural villages, while in the slums, people engaged in fewer social activities to begin with, and thus had fewer to curtail. Elderly in the rural village also had the luxury of being able to curtail economic and/or household activities. This occurred mostly because village elderly in our study lived in households where other relatives (including spouses) were able to assume responsibility for these tasks. In the slums, a much larger proportion of elderly lived either alone or with other elderly, and therefore did not have the opportunity of recruiting others to performs necessary tasks.

As has been noted previously in a study of rural villages in southern India [47], it was difficult to determine whether any of these coping strategies provided significant relief from the heat. The thermal benefit of different strategies can only be evaluated by monitoring internal body temperatures—a potential future project.

### Limitations

The length of monitoring periods is one of the most difficult issues facing studies of individually experienced heat stress. Existing studies include monitoring periods that range between 24 h (as in the current study) to multiple 7-day periods [40,61,62,63], with no consensus about which strategy is best. Essentially, it comes down to a trade-off between longer monitoring periods with fewer participants versus shorter monitoring periods with greater numbers of participants. Our decision was to emphasize the latter. Indeed, as far as we are aware, this study includes the largest number of participants of any individual-monitored heat study. This permitted us to analyze individual responses over the large temperature range that existed during the 3-month course of the study, and to generate statistically significant results using an analytical strategy involving binary logistic regressions, odds ratio analysis and chi-square determinations. Just as large samples in cross-sectional public health studies optimize the understanding of temporal variation [70], statistical analysis of the large number of different individuals exposed to the range of temperatures that existed during the 3-month period of this study provides greater understanding of the relationship between temperature and heat compared to a longitudinal study of only a week or two. Nevertheless, long-term studies of single individuals over a longer period remain a worthy goal of heat research, and would certainly improve understanding of the relationship between temperature and heat-related symptoms.

We estimated the temperatures and heat indexes associated with the probability that 80% and 90% of the participants reported being comfortable. This was based on a logistic regression relating the median 24-h experienced temperatures and heat indexes of individuals to their reports of being “comfortable/uncomfortable” during the monitoring period. Since we did not measure variables such as wet bulb globe temperature, wind velocity, and radiant temperature, or evaluate clothing insulation or metabolic rates, our estimates cannot be directly compared with determinations based on either static or adaptive thermal comfort models [94]. Nevertheless, our estimate of comfort corresponds to 80–90% temperature acceptability ranges reported in a study of rural households in Punjab [69], to the neutral temperature and comfort ranges in Indian apartments [50], and to redevelopment structures in a Mumbai slum [95], and 80% thermal acceptability of residents of Indian hostels, also based on responses to a binary question [53]. This similarity suggests that a variety of different methods (including the one we used) produce generally similar results concerning individual perceptions of comfort in hot weather in India.

## 5. Conclusions

This is the first study to focus on the heat-related symptoms and coping behaviors of elderly women and men in India. It therefore serves as a beginning to what should be many more (and more detailed) studies of elderly responses to the heat–particularly given the likely increase in hot weather and the certain increase in the number of elderly living in India. The results of this study suggest that the level of heat experienced by all the elderly participants living in Kolkata slums and in rural villages in West Bengal was well beyond the point at which heat becomes dangerous and begins to cause heat-related symptoms. Nevertheless, effective remediation will probably require different strategies in the two locations. In the urban slums, residential building materials exacerbate solar radiation [66] making alteration in housing construction one of the most effective remediation strategies for those areas. In rural areas where vegetation ameliorates heat stress to some degree, elderly women are much more likely to suffer the consequences of heat exposure than elderly men. In those areas, reduction in heat stress will likely involve behavioral changes–particularly in the domestic and economic roles played by elderly women. Thus, this study reinforces the importance of nuanced approaches in the design of heat remediation strategies in India–approaches that take into consideration the varied ways in which different sub-populations, such as the elderly, are affected by hot weather.

## Figures and Tables

**Table 1:** Characteristics of the participants.

	Kolkata Slums	Rural Villages	Chi-Square
Ages			
60–69 years	70	115	
70 years & Older	60	65	3.164 ^NS^
Gender			
Men	33	89	
Women	97	91	18.308 ***
Marital Status			
Single/Never Married	2	0	
Married	58	171	
Widow/widower	67	9	
Divorced/separated	3	0	99.548 ***
Education			
None	45	59	
Primary only	28	28	
Some Secondary	36	58	
Completed secondary	9	15	
Post-secondary	12	20	5.442 ^NS^
Currently Employed			
Yes	38	45	
No	92	135	0.689 ^NS^
Tobacco Use *			
Current/former user	38	54	
Nonuser	88	123	0.004 ^NS^
Activity			
Inactive all day	49	37	
Active primarily inmorning	20	45	
Active primarily in afternoon	34	17	
Active primarily in evening	4	0	
Active throughout the day	23	81	44.446 ***

* Not everyone responded to this question; NS = not statistically significant; *** *p* < 0.001.

**Table 2:** Temperature, humidity and heat index measurements during the study period (27 May–24 August 2019).

Easurement	Kolkata Slums			Rural Villages		
	Average 24-h		24-h	Average 24-h		24-h
	Max	Min	Median	Max	Min	Median
Temperature (°C)						
Outdoor ^1^	34.4	28.0	29.7	33.6	28.5	30.4
Indoor	34.3	30.7	32.0	33.3	28.9	30.5
Elderly Men	34.8	30.5	32.2	34.7	29.0	31.4
Elderly Women	34.7	30.7	32.2	35.0	29.0	31.6
Humidity (%)						
Outdoor ^1^	93.0	60.4	87.0	95.4	73.9	90.0
Indoor	81.7	68.6	76.5	89.5	73.3	83.7
Elderly Men	81.2	67.3	76.7	89.5	73.1	83.6
Elderly Women	81.9	69.0	76.5	89.6	73.4	83.7
Heat Index (°C)						
Outdoor ^1^	45.2	33.9	38.6	49.3	36.2	41.8
Indoor	48.7	39.3	42.9	46.7	36.8	40.6
Elderly Men	53.0	39.6	44.4	54.5	36.4	43.0
Elderly Women	53.1	40.3	44.4	57.6	36.4	44.3

^1^ Measured at Indian Meteorological Department weather stations.

**Table 3:** (**a**) Median 24-h temperatures and heat stress indexes at which there is an 80% probability of reporting comfort (*p*_comfort_ = 0.8). (**b**) Median 24-h temperatures and heat stress indexes at which there is an 90% probability of reporting comfort (*p*_comfort_ = 0.9).

**(a)**			
**Heat Measure**	**Kolkata**	**Rural Villages**	**Total**
Median 24-h			
Experienced Temperature	28.58 °C	27.82 °C	27.78 °C
(Standard Error of Estimate)	(0.23 °C)	(0.20 °C)	(0.16 °C)
Median 24-h			
Experienced Heat Index	36.89 °C	33.42 °C	34.78 °C
(Standard Error of Estimate)	(0.20 °C)	(0.21 °C)	(0.15 °C)
**(b)**			
**Heat Measure**	**Kolkata**	**Rural Villages**	**Total**
Median 24-h			
Experienced Temperature	27.67 °C	27.00 °C	26.82 °C
(Standard Error of Estimate)	(0.22 °C)	(0.19 °C)	(0.16 °C)
Median 24-h			
Experienced Temperature	35.13 °C	31.10 °C	32.67 °C
(Standard Error of Estimate)	(0.20 °C)	(0.21 °C)	(0.15 °C)

**Table 4:** Significant odds ratios reflecting gender and location differences in reported heat-related symptoms.

	**Odds Ratio (Women Relative to Men)**	**95% Confidence Intervals**		**Significance**
		**Lower**	**Higher**	
Nausea/Vomiting	4.400	1.981	10.267	*p* < 0.001
Muscle Cramps	3.054	1.903	4.901	*p* < 0.001
Disturbed Sleep	2.357	1.469	3.782	*p* < 0.001
Prickly Heat	1.767	1.105	2.824	*p* = 0.009
Dizziness	1.677	1.012	2.788	*p* = 0.022
	**Odds Ratio (Rural Villages Relative to Kolkata Slums)**	**95% Confidence Intervals**		**Significance**
		**Lower**	**Higher**	
Disturbed sleep	0.414	0.254	0.675	*p* < 0.001
Nausea/Vomiting	0.477	0.254	0.894	*p* = 0.011
Muscle Cramps	0.536	0.338	0.849	*p* = 0.004
Prickly heat	2.072	1.298	3.309	*p* = 0.010

**Table 5:** Heat Index Levels Experienced by Individuals who Reported Different Numbers of Heat-Related Symptoms.

Heat Index	0–3 Symptoms			4–7 Symptoms			8–11 Symptoms			F
Measure	N	Mean	S.E.	N	Mean	S.E.	N	Mean	S.E.	
**Median 24-h Experienced HI (°C)**										
Kolkata Slums (values adjusted to median 24-h indoor HI = 42.7 °C)										
Women	11	44.3	0.4	46	43.6	0.2	40	44	0.2	1.842 ^NS^
Men	10	42.7	0.4	14	43.8	0.4	9	43.9	0.5	2.662 ^NS^
F		4.694 *			0.179 ^NS^			0.043 ^NS^		
Rural Villages (values adjusted to median 24-h outdoor HI = 41.3 °C)										
Women	8	44	0.9	55	43.9	0.3	28	44.5	0.6	0.818 ^NS^
Men	13	42.1	0.7	65	42.5	0.3	11	43.1	0.7	0.390 ^NS^
F		2.874 ^NS^			10.774 **			2.988 ^NS^		
**Experienced HI at 1 P.M. (°C)**										
Kolkata Slums (values adjusted to indoor HI at 1 P.M. = 47.1 °C)										
Women	11	47.4	0.9	46	48	0.4	40	49.4	0.5	3.303 *
Men	10	48.4	0.9	14	48.4	0.8	9	50.3	1	1.481 ^NS^
F		1.273 ^NS^			0.284 ^NS^			0.489 ^NS^		
Rural Villages (values adjusted to outdoor HI at 1 P.M. = 47.1 °C)										
Women	8	46.9	1.6	55	50.3	0.6	28	51.2	0.8	3.092 *
Men	13	46.6	1.2	65	48.2	0.5	11	48.2	1.3	0.875 ^NS^
F		0.141 ^NS^			5.863 *			5.050 *		

^NS^ Not Statistically Significant; * *p* < 0.05; ** *p* < 0.001.

**Table 6:** Summary of significant demographic, social-cultural and experienced heat variables associated with coping strategies, determined by binary logistic regression. Predictors are arranged according to Wald value (largest = first predictor, etc.).

Coping Strategy	First Predictor	Second Predictor	Third Predictor	Fourth Predictor	Fifth Predictor	Nagelkerke R^2^
Use Electric Fan In Sleeping Area	Walls made of Cement	Personal HI Overnight	Post-Secondary Education			0.179
Rest	Walls made of Brick	Excessive Thirst	Number of Rooms ‘in Dwelling			0.154
Drink Water	Excessive Thirst	Married				0.309
Move to Cooler Area	Rural Villages	Roof made of Cement	Dizziness	Nausea/Vomiting		0.535
Use Hand Fan	Personal HI Afternoon	Excessive Sweating	Fatigue/Weakness			0.122
Alter/Reduce Social Activities	Rural Villages	Walls made of brick	Personal HI Afternoon	Post-Secondary Education	Active All Day	0.622
Change/Remove Clothing	Active in Afternoon	Men	Roof made of asbestos sheets	Number of rooms in dwelling		0.175
Add Food Items	Kolkata Slums	Under 70 Years	No Education	Dizziness	Prickly Heat	0.367
Avoiding/Altering Activities	Rural Villages	Walls made of mud	Uncomfortable in Heat	Excessive Thirst	Number of rooms in dwelling	0.585
Take Shower or Bath	Rural Villages	Roof made of Thatch	Secondary Ed	Uncomfortable	Walls made of Mud	0.444
Delete Food Items	Rural Villages	Personal HI, Morning	Disturbed Sleep Overnight	Personal HI,	Prickly Heat	0.297

Note: Analysis based on individuals for whom all data exist.

## Data Availability

The data set, on which the findings of the study are based, is not publicly available because it contains indirect identifiers that could compromise the anonymity of research participants. However, concatenated data related to buildings, building materials and indoor heat measurements are available in [66] (as supplements), and concatenated data related to individual experienced heat conditions are available in [83] (as supplements). Concatenated data related to heat-related symptoms and heat-coping strategies are available in the supplements to this article.

## Supplementary Materials

The following are available online at https://www.mdpi.com/article/10.3390/ijerph191912446/s1, Figure S1. The percent of elderly individuals who reported different numbers of heat-related symptoms by location (upper panel) and by gender (lower panel). File S1. Instrumentation Specifications. File S2. Heat Index Formula. File S3. Questionnaire. File S4. Details of Binary Logistic Regression Model. File S5. Chi-Square analysis for gender & location differences of symptoms & coping behaviors. File S6. Odds Ratios Relating Characteristics to Symptoms. File S7. Symptom Odds—Variance Explained by HI. File S8. Odds Ratios Relating Comorbidities with Symptoms. File S9. Odds Ratios Relating characteristics to Coping Strategies. File S10. Binary Logistic Regressions for Coping Behavior. References [71,96,97] are cited in the Supplementary Materials.
