# Supplementary material for: Heat Exposure, Heat-Related Symptoms and Coping Strategies among Elderly Residents of Urban Slums and Rural Vilages in West Bengal, India
Source: Int J Environ Res Public Health. 2022 Sep 29;19(19):12446. doi: 10.3390/ijerph191912446 (PMC9564637; doi:10.3390/ijerph191912446)
Supplement: Supplementary file 1 [file ijerph-19-12446-s001.zip › Supplemental File S1. Instrumentation Specifications.pdf]

**Supplemental File S1**. Instrument Specifications

| Measurement | Instrument Used | |
|---|---|---|
| | Kestrel D-2 | Kestrel 5400 |
| AMBIENT TEMPERATURE | | |
| Sensor Accuracy (+/-) | 0.9 °F, 0.5 °C | 0.9 °F, 0.5 °C |
| Resolution | 0.1 °C, 0.1 °F | 0.1 °C, 0.1 °F |
| Specification Range | -10 to 55 °C,<br>14 to 131°F | -29.0 to 70.0 °C,<br>-20.0 to 158.0 °F |
| RELATIVE HUMIDITY | | |
| Sensor Accuracy (+/-) | 2% RH | 2% RH |
| Resolution | 0.1 % RH | 0.1 % RH |
| Specification Range | 10 to 90% | 10 to 90% |
| HEAT INDEX | | |
| Sensor Accuracy (+/-) | 4.0°C ,7.1°F | 4.0°C ,7.1°F |
| Resolution | 0.1 °C, 0.1 °F | 0.1 °C, 0.1 °F |
