# Supplementary material for: Heat Exposure, Heat-Related Symptoms and Coping Strategies among Elderly Residents of Urban Slums and Rural Vilages in West Bengal, India
Source: Int J Environ Res Public Health. 2022 Sep 29;19(19):12446. doi: 10.3390/ijerph191912446 (PMC9564637; doi:10.3390/ijerph191912446)
Supplement: Supplementary file 1 [file ijerph-19-12446-s001.zip › Supplemental File S10. Binary Logistic Regressions for Coping Behavior.pdf]

Supplemental File S10. Binary Logistic regressions indicating heat-related symptoms, participant characteristics, individual experienced HI and dwelling characteristics significantly related to coping strategies

	B	Standard Error	Wald	Significance (p value)	Exp(B)	95% CI of Exp(B) Lower	95% CI of Exp(B) Upper
**Use Electric Fan in Sleeping Area (yes = 279; no = 23)**							
Constant	-3.309	0.352	88.392	< 0.001	0.037		
Dwelling Walls of Cement	1.659	0.468	12.556	< 0.001	5.252	2.098	13.144
Personal HSI, Overnight	-0.541	0.249	4.731	0.030	0.582	0.358	0.948
Post-Secondary Ed	1.247	0.648	3.705	0.054	3.479	0.947	12.383
Nagelkerke R^2 = 0.179; -2 log likelihood = 139.132							
"yes" responses correctly classified = 100%; "no" responses correctly classified = 4.3%							
**Resting (yes = 264; no = 38)**							
Constant	-4.996	0.891	31.444	< 0.001	0.007		
Dwelling Walls of Brick	2.422	0.746	10.051	0.002	11.269	2.521	50.372
Excessive Thirst	1.226	0.404	9.219	0.002	3.407	1.544	7.516
Rooms in Dwelling	0.345	0.161	4.585	0.032	1.411	1.030	1.934
Nagelkerke R^2 = 0.154; -2 log likelihood = 202.617							
"yes" responses correctly classified = 100%; "no" responses correctly classified = 2.6%							
**Drink Water (yes = 261; no = 40)**							
Constant	-3.100	0.322	92.583	< 0.001	0.045		
Excessive Thirst	2.571	0.381	43.167	< 0.001	13.081	6.075	28.037
Married vs Unmarried	1.072	0.399	7.225	0.007	2.921	1.337	6.384
Nagelkerke R^2 = 0.309; -2 log likelihood = 180.408							
"yes" responses correctly classified = 97.7%; "no" responses correctly classified = 32.5%							
**Move to a cooler area (yes = 214; no = 87)**							

Constant	-4.754	0.621	58.684	< 0.001	0.009		
Rural Villages vs Kolata Slums	3.816	0.437	76.360	< 0.001	45.404	19.294	106.847
Dwelling Roof of Cement	1.231	0.410	9.019	0.003	3.424	1.534	7.646
Dizziness	0.888	0.370	5.779	0.016	2.431	1.178	5.016
Nausea/Vomiting	1.054	0.473	5.969	0.026	2.870	1.136	7.255

Nagelkerke R^2 = 0.535; -2 log likelihood = 220.667

“yes” responses correctly classified = 84.5%; “no” responses correctly classified = 79.3%

Use a Hand Fan (yes = 207; no = 92)

Constant	-1.213	0.444	49.186	< 0.001	0.297		
Personal HSI, Afternoon	0.551	0.143	14.919	< 0.001	1.735	1.312	2.294
Excessive Sweating	0.787	0.312	6.349	0.012	2.198	1.191	4.054
Fatigue/Weakness	0.690	0.305	5.104	0.024	1.993	1.096	3.626

Nagelkerke R^2 = 0.122; -2 log likelihood = 339.902

“yes” responses correctly classified = 94.7%; “no” responses correctly classified = 24.2%

Alter Social Activities (yes = 190; no = 108)

Constant	-2.336	0.316	55.541	< 0.001	0.037		
Rural Villages vs Kolata Slums	4.168	0.459	82.408	< 0.001	64.585	26.261	158.863
Dwelling Walls of Brick	1.740	0.614	8.032	0.005	5.695	1.170	18.965
Higher Education	1.524	0.583	6.835	0.009	4.593	1.465	14.402
Active All Day	1.127	0.470	5.739	0.017	3.086	1.227	7.759
Personal HSI, Afternoon	-0.417	0.185	5.049	0.025	1.517	1.055	2.183

Nagelkerke R^2 = 0.622; -2 log likelihood = 209.460

“yes” responses correctly classified = 86.8%; “no” responses correctly classified = 85.2%

Change or Remove Clothing (yes = 186; no = 114)

Constant	-3.712	0.881	17.767	< 0.001	0.024		
Active in the afternoon	1.259	0.349	13.034	< 0.001	3.564	1.788	6.981

Men compared to women	0.892	0.278	10.298	0.001	2.441	1.415	4.210
Dwelling Roof of Asbestos	0.772	0.293	6.959	0.008	2.164	1.219	3.840
Number of rooms in dwelling	0.231	0.118	3.815	0.050	1.260	1.000	1.589

Nagelkerke R^2 = 0.175; -2 log likelihood = 355.285

"yes" responses correctly classified = 87.1%; "no" responses correctly classified = 32.7%

Adding Food Considered to be Heat-Appropriate to Diet (yes = 156; no = 146)

Constant	-4.481	0.682	43.176	< 0.001	0.011		
Kolkata Slums vs Rural Villages	1.790	0.304	27.537	< 0.001	5.987	3.069	11.682
60 – 69 years vs $\geq$ 70 years	1.248	0.296	17.754	< 0.001	3.483	1.949	6.223
No Education	1.230	0.304	16.352	< 0.001	3.421	1.885	6.209
Dizziness	1.032	0.308	11.257	< 0.001	2.807	1.536	5.130
Prickly Heat	0.946	0.306	9.532	0.002	2.575	1.413	4.694
Dwelling Roof of Asbestos	0.932	0.339	7.568	0.006	2.540	1.307	4.935
Currently Employed	0.815	0.322	6.569	0.010	2.283	1.214	4.291
Muscle Cramps	0.688	0.293	5.494	0.019	1.990	1.119	3.537
Excessive Thirst	0.871	0.385	5.115	0.024	2.389	1.123	5.082

Nagelkerke R^2 = 0.365; -2 log likelihood = 320.859

"yes" responses correctly classified = 72.9%; "no" responses correctly classified = 68.5%

Avoiding or Reducing Household/Economic Activities (yes = 130; no = 168)

Constant	-3.481	0.576	18.553	< 0.001	0.084		
Rural Villages vs Kolkata Slims	4.761	0.572	69.317	< 0.001	116.887	38.106	358.457
Dwelling walls of mud	1.135	0.437	6.756	0.009	3.113	1.322	7.327
Uncomfortable in Heat	1.080	0.435	6.168	0.013	2.945	1.256	6.907
Excessive Thirst	0.908	0.426	4.540	0.033	2.480	1.075	5.718
Number of rooms in dwelling	0.330	0.168	3.847	0.050	1.392	1.000	1.963

Nagelkerke R^2 = 0.585; -2 log likelihood = 236.635

"yes" responses correctly classified = 90.8%; "no" responses correctly classified = 77.2%

Take a Bath, Shower or Sponge Bath (yes = 93; no = 204)

Constant	-3.606	0.989	13.293	< 0.001	0.027		
Rural Villages vs Kolkata Slums	3.544	0.553	41.109	< 0.001	35.594	11.710	102.200
Dwelling Roof of Thatch	2.105	0.757	7.088	0.008	7.503	1.702	33.080
Secondary Education	1.430	0.605	5.582	0.018	4.179	1.276	13.689
Uncomfortable in Heat	1.050	0.450	5.446	0.020	2.859	1.183	6.907
Dwelling Walls of Mud	0.846	0.411	4.236	0.040	2.330	1.041	5.215

Nagelkerke R^2 = 0.444; -2 log likelihood = 256.089

“yes” responses correctly classified = 80.6%; “no” responses correctly classified = 76.8%

Deleting Food Considered to be Heat-Inappropriate from Diet (yes = 73; no = 229)

Constant	-1.228	0.367	11.166	< 0.001	0.283		
Rural Villages vs Kolkata Slums	2.828	0.492	33.078	< 0.001	16.914	6.452	44.341
Personal HSI, Morning	0.583	0.190	8.010	0.005	1.713	1.180	2.487
Disturbed Sleep	0.852	0.338	6.339	0.012	2.344	1.208	4.549
Personal HSI, Overnight	-0.429	0.179	5.757	0.016	0.651	0.458	0.924
Dwelling Roof of Asbestos	0.838	0.349	5.748	0.017	2.311	1.165	4.583
No Education	0.784	0.344	5.207	0.022	2.190	1.117	4.294
Prickly Heat	0.816	0.353	5.35	0.021	2.262	1.133	4.518

Nagelkerke R^2 = 0.297; -2 log likelihood = 271.436

“yes” responses correctly classified = 27.8%; “no” responses correctly classified = 93.4%
