# Supplementary material for: Heat Exposure, Heat-Related Symptoms and Coping Strategies among Elderly Residents of Urban Slums and Rural Vilages in West Bengal, India
Source: Int J Environ Res Public Health. 2022 Sep 29;19(19):12446. doi: 10.3390/ijerph191912446 (PMC9564637; doi:10.3390/ijerph191912446)
Supplement: Supplementary file 1 [file ijerph-19-12446-s001.zip › Supplemental File S2. Heat Index Formula.pdf]

Supplemental File S2. Formula for the computation of Heat Index (HI).

$$HI = C_1 + C_2T + C_3R + C_4TR + C_5T^2 + C_6R^2 + C_7T^2R + C_8TR^2 + C_9T^2R^2$$

Where:

HI = heat index (in degrees Celsius)
T = ambient dry-bulb temperature (in degrees Celsius)
R = relative humidity (percentage value between 0 and 100)
C_1 = -8.78469475556
C_2 = 1.61139411
C_3 = 2.33854883889
C_4 = -0.14611605
C_5 = -0.012308094
C_6 = -0.0164248277778
C_7 = 0.002211732
C_8 = 0.00072546
C_9 = -0.000003582

Reference: Rothfusz, L.P. 1990. The Heat Index "Equation" (or, More Than You Ever Wanted to Know About Heat Index). Scientific Services Division, NWS Southern Region Headquarters, Fort Worth, TX, Technical Attachment SR 90-23.
