# Supplementary material for: Heat Exposure, Heat-Related Symptoms and Coping Strategies among Elderly Residents of Urban Slums and Rural Vilages in West Bengal, India
Source: Int J Environ Res Public Health. 2022 Sep 29;19(19):12446. doi: 10.3390/ijerph191912446 (PMC9564637; doi:10.3390/ijerph191912446)
Supplement: Supplementary file 1 [file ijerph-19-12446-s001.zip › Supplemental File S3. Questionnaire.pdf]

Supplemental File S3.

PARTICIPANT QUESTIONNAIRE FOR STUDY OF ELDERLY RESIDENTS IN WEST BENGAL

Part 1. General Information about Person Interviewed

1. Date of Interview:

2. Name:

3. Temporary ID No:

4. Name of the interviewer:

5. Name town/village:

6. Type of ecological setting: rural village/urban slum

7. Age:

8. Age group: 60–64 65-69 70–74 75–79 > 80

10. Sex: Male Female

11. Education: None Primary Some Secondary Completed Secondary University Polytechnic/Diploma

12. Smoking: Smoker Non Smoker Ex-smoker

13. Consuming alcohol: Yes No Ex

14. Which of the following best describes your marital status?

    Single

    Married

    Divorced

    Widowed

15. How many people do you live with? __________________

Describe relationships:

Part 2. Questions Concerning Work

1. Are you currently employed? Yes / No

   a. If yes, how many hours per day do you work usually? __________________

2. Type of work: ____

   a. Is your work: Light Moderate Heavy Very Heavy

3. Is your work indoors or outdoors?

   a. If indoors, is the place you work well-ventilated? Yes / No

4. If you are not employed, what tasks/chores do you do in the morning?

   Specify:

   a. How many hours/day, do you perform these tasks/chores?

5. If you are not employed, what tasks/chores do you do in the afternoon?

   Specify:

   a. How many hours/day, do you perform these tasks/chores?

6. If you ae not employed, what tasks/chores do you do in the evening?

   Specify:

   a. How many hours/day, do you perform these tasks/chores?

Part 3. Questions about housing

1. How many rooms in your house? __________________

2. Do you possess any of the following electrical devices?

   a. fans

   b. air conditioner

   c. Television

   d. Radio

   e. Microwave

f. Electric heater

g. Electric kettle

h. Refrigerator

i. Others, please specify

3. Type of fuel used for cooking

a. Firewood

b. Crop Residue

c. Dung

e. Charcoal/Coal

f. Kerosene

g. Liquid petroleum gas

h. electricity

4. Location of cookstove

a. Inside

b. Outside

Part 5. Questions Concerning Sleeping Habits during Summer Months

1. How many hours do you sleep during summer months (overnight and during day combined)?

a. 1–2 h

b. 2–4 h

c. 4–6 h

d. 6-8 h

e. More than 8 h

2. Where do you usually sleep during the summer month?

Room Terrace/porch Balcony Other common area.

3. What is the reason for your sleeping in a specific area?

4. If sleeping inside,

Are there doors in your sleeping location? Yes/No

Are they open or closed at night? Open/Closed

Are there windows in your sleeping location? Yes/No

Are they open or closed at night? Open/Closed

5. In your sleep location, do you have:

a. Fan Yes/No

b. Air Conditioner Yes/No

c. Hand Fan Yes/No

d. Cooler Yes/No

e. Natural Breeze Yes/No

6. Does the electricity go off during the time you are sleeping? Yes/No

a. If so, how much time is spent without fan/air conditioner/cooler? ________________

Part 6. Questions in Relation to Heat Exposure at Work/at home

1. Are you comfortable with the ambient temperature over the past 24 hours? Yes/ No

2. Has the heat been a problem to you over the past 24 hours? Yes/No

3. How many months do you feel hot /uncomfortably hot in the workplace/or at home?

1–3 months 4–6 months 7–9 months 9–12 months Never

4. Describe how bad the heat stress can be in the hot season.

Extremely bad Very bad Bad Manageable No stress at all

Part 7. Questions Concerning Impacts of Heat on Health

1. Have you ever had these symptoms at work/at home.

a. Excessive sweating: Yes/ No

b. Muscle cramps: Yes/ No

c. Excessive Thirst: Yes / No

d. Tiredness/weakness: Yes/ No

e. Dizziness: Yes/No

f. Headache: Yes/ No

g. Nausea or vomiting: Yes/No

h. Fainting: Yes/No

i. Prickly heat: Yes/No

2. Do you have any of these existing illness or health problems:

None Diabetes Hypertension Heart Problems Respiratory illness

3. Have you even been admitted to hospital/medical center due to heat-related problems? Yes/No

Part 8. Questions Concerning Coping Mechanisms

1. How do you manage heat exposure during hot days?

a. Take rest Yes/No

b. Change/remove clothing Yes/No

c. Use hand fan Yes/No

d. Take shower, bath, or sponge bath Yes / No

e. Move to a shaded/cooler environment Yes/No

g. Avoid/reduce household/economic activities? Yes/No

h Aoid/reduce social activities Yes/No

i. Use of fans in sleeping area at night Yes/No

j. Drink water Yes/No

I. Source: Tube well / Tap

II. Are these in working condition: Yes/No

III. If, No, how long they ae not working?

IV. How far is the source of water?.............. meter

Part 9. Questions Concerning Food Avoidance/ Preference during Summer Months

1. Do you avoid or consume any specific type of food during summer months? Yes/ No

   a. What types of foods do you consume because of the heat?

      1. ____________________
      2. ____________________
      3. ____________________
      4. ____________________

   b. If so, what are the foods you avoid because of the heat?

      1. ____________________
      2. ____________________
      3. ____________________
      4. ____________________
