# Supplementary material for: Heat Exposure, Heat-Related Symptoms and Coping Strategies among Elderly Residents of Urban Slums and Rural Vilages in West Bengal, India
Source: Int J Environ Res Public Health. 2022 Sep 29;19(19):12446. doi: 10.3390/ijerph191912446 (PMC9564637; doi:10.3390/ijerph191912446)
Supplement: Supplementary file 1 [file ijerph-19-12446-s001.zip › Supplemental File S4. Details of Binary Logistic Regression Model.pdf]

Supplemental File S4. Details of binary logistic regression analysis for determining the likelihood of reporting heat-related symptoms

In order to include information on the 24-hour pattern of IEHI and to overcome problems of collinearity among consecutive hourly HI measurements, a principal component analysis was used to reduce hourly IEHI data into a set of unrelated, orthogonal components. The number of components used for further analysis was determined by reference to eigenvalues from randomly generated correlation matrices [Patil et al 2017]. The number of factors retained were those where the eigenvalues generated by the PCA were larger than the corresponding random eigenvalues [Horn 1965]. Three principal components (PC's) were identified by this process and entered as an independent variable. Additional independent variables entered into the equation were: binary (yes/no) reports provided by participants concerning the existence of each of the heat-related symptoms, age (60 – 69 years vs 70 years and older), gender, location, marital status (married vs single/divorced/widowed) employment (currently employed versus retired), tobacco use (non-user vs current/former user), education, activity, and dwelling characteristics (number of co-inhabitants, and number of rooms in the dwelling, dwelling wall materials, dwelling roof material). Education was recoded as four binary variables: (1) no education compared to some education, (2) highest education = primary level compared to other education categories, (3) highest education = secondary level compared to other education categories, (4) highest education = post-secondary education compared to other education categories. Responses to activity questions were transformed into four binary variables: (1) inactive all day versus other activity categories, (2) active all day versus other activity categories, (3) active in the morning versus other activity categories and (4) active in the afternoon versus other activity categories. Dwelling roof material was recoded as: (1) cement versus asphalt sheet or thatch roofs, (2) asphalt sheet versus cement or thatch roofs, and (3) thatch versus cement or asphalt sheet roofs. Dwelling wall material was recoded as: (1) cement versus brick or mud walls, (2) brick versus cement or mud walls, and (3) mud versus cement or brick walls. A Wald test was used to determine if the maximum likelihood estimation of a coefficient differed significantly from a null hypothesis of 0, and thus significantly improved the model fit.

References:

Patil, V.H.; Singh, N.H.; Mishra, S.; Donavan, T., 2017. Parallel Analysis Engine to Aid in Determining Number of Factors to Retain using R [Computer software], available from https://analytics.gonzaga.edu/parallelengine/.

Horn, J.L., 1965. A Rationale and Test for the Number of Factors in Factor Analysis. Psychometrika, 30:179-185. https://doi.org/10.1007/BF02289447
