# Supplementary material for: Heat Exposure, Heat-Related Symptoms and Coping Strategies among Elderly Residents of Urban Slums and Rural Vilages in West Bengal, India
Source: Int J Environ Res Public Health. 2022 Sep 29;19(19):12446. doi: 10.3390/ijerph191912446 (PMC9564637; doi:10.3390/ijerph191912446)
Supplement: Supplementary file 1 [file ijerph-19-12446-s001.zip › Supplemental File S5. Chi-Square analysis for gender & location differences of symptoms & coping behaviors.pdf]

Supplemental File S5. Chi-square for differences in heat-related symptoms and heat-related coping behaviors by location and gender.

Condition	Kolkata Slum Men N	Kolkata Slum Women N	Rural Villages Men N	Rural Villages Women N	Chi-Square
Heat-related Symptoms: Uncomfortable in heat					
Yes	23	80	73	79	χ^2 (location) = 1.406^{NS}
No	10	17	16	12	χ^2 (sex) = 1.756^{NS}
Heat-related Symptoms: Disturbed sleep					
Yes	23	73	38	59	χ^2 (location) = 12.795***
No	10	24	51	32	χ^2 (sex) = 12.864***
Heat-related Symptoms: Excessive sweating					
Yes	24	83	70	75	χ^2 (location) = 0.152^{NS}
No	9	14	19	16	χ^2 (sex) = 2.379^{NS}
Heat-related Symptoms: Muscle cramps					
Yes	12	70	34	52	χ^2 (location) = 7.117**
No	21	27	55	39	χ^2 (sex) = 22.032***
Heat-related Symptoms: Excessive thirst					
Yes	25	80	73	76	χ^2 (location) = 0.206^{NS}
No	8	17	16	15	χ^2 (sex) = 0.351^{NS}
Heat-related Symptoms: Fatigue/weakness					
Yes	21	74	69	73	χ^2 (location) = 1.418^{NS}
No	12	23	20	18	χ^2 (sex) = 0.803^{NS}
Heat-related Symptoms: Dizziness					

Yes	8	35	22	35	χ^2 (location) = 1.778^{NS}
No	25	62	67	66	χ^2 (sex) = 3.236*
Heat-related Symptoms: Nausea/vomiting					
Yes	5	22	2	18	χ^2 (location) = 5.474*
No	28	75	87	73	χ^2 (sex) = 13.889***
Heat-related Symptoms: Fainting					
Yes	6	3	5	4	χ^2 (location) = 0.510^{NS}
No	27	94	84	87	χ^2 (sex) = 3.790*
Heat-related Symptoms: Headache					
Yes	8	35	22	25	χ^2 (location) = 1.778^{NS}
No	25	62	67	66	χ^2 (sex) = 1.927^{NS}
Heat-related Symptoms: Prickly heat (heat rash)					
Yes	13	55	53	72	χ^2 (location) = 9.434**
No	20	42	36	19	χ^2 (sex) = 5.700*
Coping behavior: take rest					
Yes	30	79	77	86	χ^2 (location) = 3.159^{NS}
No	3	18	12	5	χ^2 (sex) = 0.000^{NS}
Coping behavior: change or remove clothing					
Yes	24	41	66	59	χ^2 (location) = 11.025***
No	7	56	23	32	χ^2 (sex) = 14.740***
Coping behavior: drink water					
Yes	27	79	79	84	χ^2 (location) = 6.060*

No	6	18	9	7	χ^2 (sex) = 0.053^{NS}
Coping behavior: use hand fan					
Yes	20	68	66	59	χ^2 (location) = 0.041^{NS}
No	12	28	23	31	χ^2 (sex) = 0.270^{NS}
Coping behavior: Use electric fan in sleeping area					
Yes	32	94	78	81	χ^2 (location) = 7.512**
No	1	3	11	10	χ^2 (sex) = 0.852^{NS}
Electricity goes off during sleep					
Yes	7	28	89	91	χ^2 (location) = 188.508***
No	26	68	0	0	χ^2 (sex) = 7.903**
Coping behavior: Natural breeze in sleeping area					
Yes	16	41	57	50	χ^2 (location) = 7.268**
No	17	54	32	39	χ^2 (sex) = 3.178^{NS}
Coping behavior: take shower, bath or sponge bath					
Yes	2	2	44	45	χ^2 (location) = 75.591***
No	30	92	45	45	χ^2 (sex) = 5.358*
Coping behavior: move to shaded/cooler environment					
Yes	10	41	81	87	χ^2 (location) = 108.852***
No	23	56	7	4	χ^2 (sex) = 1.809^{NS}
Coping behavior: avoid activities or reduce activity level					
Yes	2	4	61	65	χ^2 (location) = 136.737***

No	31	93	26	24	χ^2 (sex) = 7.055**
Coping behavior: reduce social activities					
Yes	9	24	78	84	χ^2 (location) = 143.735***
No	24	73	8	6	χ^2 (sex) = 7.418**
Coping behavior: add food items considered appropriate for the heat					
Yes	21	74	31	38	χ^2 (location) = 36.570***
No	12	23	58	53	χ^2 (sex) = 8.533**
Coping behavior: omit food items not considered appropriate for the heat					
Yes	2	13	29	31	χ^2 (location) = 19.550***
No	31	84	60	60	χ^2 (sex) = 0.162^{NS}
