# Supplementary material for: Heat Exposure, Heat-Related Symptoms and Coping Strategies among Elderly Residents of Urban Slums and Rural Vilages in West Bengal, India
Source: Int J Environ Res Public Health. 2022 Sep 29;19(19):12446. doi: 10.3390/ijerph191912446 (PMC9564637; doi:10.3390/ijerph191912446)
Supplement: Supplementary file 1 [file ijerph-19-12446-s001.zip › Supplemental File S6. Odds Ratios Relating Characteristics to Symptoms.pdf]

Supplemental File S6. Odds ratios relating different participant characteristics to reported heat-related symptoms

	Excessive Thirst	Excessive Sweating	Fatigue Weakness	Disturbed Sleep	Prickly Heat
Age (reference = 60 – 69 years)					
70 years & older	**0.6178**	**0.3337**	0.8906	1.1327	0.7604
Location (reference = Kolkata slums)					
Rural Villages	1.1444	0.8905	1.3767	**0.4139**	**2.0722**
Gender (reference = men)					
Women	1.1939	1.5688	1.2748	**2.3571**	**1.7665**
Marital Status (reference = married)					
Not currently married	**0.5689**	0.7407	1.1063	**2.0623**	0.7859
Education (reference = none)					
Primary only	0.9517	1.0805	**0.3767**	0.6869	**0.5419**
Some secondary	0.8636	0.9371	0.8837	0.6847	**0.3985**
completed secondary	2.2759	0.7862	0.6280	0.7407	1.3325
post-secondary	0.5287	**0.4552**	**0.3996**	**0.3922**	**0.2400**
Currently employed (reference = no)					
Yes	0.6560	08558	1.1547	0.9540	0.7756
Tobacco use (reference category = nonuser)					
Current/former tobacco user	0.9691	0.6955	0.8120	1.3015	0.7022
Activity (reference = inactive)					
Morning active	0.6478	0.8187	**2.1273**	0.7831	**1.8670**
Afternoon active	0.7883	0.5395	1.2667	1.1852	1.2447
Active all day	**0.5165**	**0.3571**	1.5261	0.9481	**1.6359**

	Muscle Cramps	Dizziness	Headache	Nausea Vomiting	Fainting
Age (reference = 60 – 69 years)					
70 years & older	**0.6581**	1.2206	0.8607	0.9049	1.5172
Location (reference = Kolkata slums)					
Rural Villages	**0.5355**	0.8604	0.7150	**0.4769**	0.7076
Gender (reference = men)					
Women	**3.0540**	**1.6771**	1.4375	**4.4402**	**0.3903**
Marital Status (reference = married)					
Not currently married	**2.9525**	1.2954	1.5359	**2.2000**	0.7978
Education (reference = none)					
Primary only	0.7750	**0.5511**	1.0572	0.9111	1.0659
Some secondary	0.6806	**0.4444**	0.6291	**0.3947**	0.9448
completed secondary	**0.4464**	0.5253	0.6863	0.3388	0.5774
post-secondary	**0.4861**	**0.3457**	0.8056	0.3856	0.4470
Currently employed (reference = no)					
Yes	1.0013	0.8523	0.8433	0.5142	1.8086
Tobacco use (reference category = nonuser)					
Current/former tobacco user	0.7402	0.7046	**0.5842**	**0.1776**	1.9143
Activity (reference = inactive)					
Morning active	0.9993	1.3148	0.8736	**0.3646**	1.0623
Afternoon active	1.3016	1.0286	1.1670	1.0671	0.8100
Active all day	1.0630	0.7482	**0.5273**	0.8949	0.9918

Statistically significant odds ratios (p < 005) indicated in bold
