# Supplementary material for: Heat Exposure, Heat-Related Symptoms and Coping Strategies among Elderly Residents of Urban Slums and Rural Vilages in West Bengal, India
Source: Int J Environ Res Public Health. 2022 Sep 29;19(19):12446. doi: 10.3390/ijerph191912446 (PMC9564637; doi:10.3390/ijerph191912446)
Supplement: Supplementary file 1 [file ijerph-19-12446-s001.zip › Supplemental File S7. Symptom Odds - Variance Explained by HI.pdf]

Supplemental File S7. Binary Logistic regressions indicating predictability of heat-related symptoms based on principle components of individually experienced HI.

	B	Standard Error	Wald	Significance (p value)	Exp(B)	95% CI of Exp(B) Lower	Upper
**Excessive Thirst (yes = 253; no = 56)**							
No significant individually-experienced HI principle component predictors							
**Excessive Sweating (yes = 251; no = 58)**							
Constant	-1.501	0.150	99.809	< 0.001	0.223		
Principle Component 3 (Afternoon IEHI)	-0.345	0.153	5.129	0.024	0.707	0.525	0.955
Nagelkerke R^2 = 0.028; -2 log likelihood = 293.095							
**Fatigue/Weakness (yes = 236; no = 73)**							
Constant	-1.205	0.138	76.672	< 0.001	0.300		
Principle Component 3 (Afternoon IEHSI)	-0.353	0.141	6.285	0.012	0.703	0.533	0.926
Nagelkerke R^2 = 0.032; -2 log likelihood = 331.317							
**Disturbed Sleep (yes = 192; no = 117)**							
No significant individually-experienced HI principle component predictors							
**Prickly Heat (yes = 192; no = 117)**							
Constant	-0.522	0.121	18.669	< 0.001	0.593		
Principle Component 2 (Morning IEHI)	-0.369	0.126	8.651	0.003	0.691	0.540	0.884
Principle Component 1 (Overnight IEHI)	0.274	0.123	4.934	0.026	1.316	1.033	1.676
Nagelkerke R^2 = 0.060; -2 log likelihood = 396.070							

Muscle Cramps (yes = 167; no = 142)

No significant individually-experienced HI principle component predictors

Dizziness (yes = 176; no = 133)

Constant	-0.287	0.117	6.045	0.014	0.750		
Principle Component 3 (Afternoon IEHI)	-0.261	0.118	4.868	0.027	0.770	0.611	0.971
Principle Component 1 (Overnight IEHI)	-0.255	0.118	4.664	0.031	0.775	0.615	0.977

Nagelkerke R^2 = 0.042; -2 log likelihood = 412.629

Headache (yes = 90; no = 219)

Constant	0.909	0.117	50.845	< 0.001	2.481		
Principle Component 1 (Overnight IEHI)	-0.305	0.131	5.456	0.019	0.737	0.571	0.952

Nagelkerke R^2 = 0.026; -2 log likelihood = 367.176

Nausea/Vomiting (yes = 47; no = 262)

Constant	1.860	0.179	108.595	< 0.001	6.426		
Principle Component 1 (Overnight IEHI)	-0.526	0.175	9.013	0.003	0.591	0.419	0.823
Principle Component 3 (Afternoon IEHI)	-0.400	0.170	5.537	0.019	0.670	0.480	0.935

Nagelkerke R^2 = 0.082; -2 log likelihood = 248.574

Fainting (yes = 18; no = 291)

No significant individually-experienced HI principle component predictors

---

IEHI = Individually-experienced Heat Index
