# Supplementary material for: Heat Exposure, Heat-Related Symptoms and Coping Strategies among Elderly Residents of Urban Slums and Rural Vilages in West Bengal, India
Source: Int J Environ Res Public Health. 2022 Sep 29;19(19):12446. doi: 10.3390/ijerph191912446 (PMC9564637; doi:10.3390/ijerph191912446)
Supplement: Supplementary file 1 [file ijerph-19-12446-s001.zip › Supplemental File S8. Odds Ratios Relating Comorbidities with Symptoms.pdf]

Supplemental File S8. Odds of reporting heat-related symptoms among individuals with different chronic conditions.

Heat-Related Symptoms	None	Hypertension	Heat Disease	Diabetes	Respiratory Diseases
Discomfort	0.743	1.330	1.552	1.330	1.109
Excessive Thirst	1.066	1.017	1.812	0.785	0.864
Excessive Sweating	**0.504***	1.225	0.699	**5.341***	0.699
Fatigue/Weakness	**0.621***	1.152	2.693	0.932	2.356
Disturbed Sleep	**0.438***	1.139	4.857	1.376	**3.036***
Prickly Heat	0.698	0.974	1.164	1.582	1.396
Muscle Cramps	0.751	1.255	0.502	1.027	0.989
Dizziness	**0.568***	1.272	1.313	1.143	1.313
Headache	0.918	1.054	0.952	1.032	0.953
Nausea/Vomiting	0.898	0.997	0.719	1.294	1.150
Fainting	**0.441***	1.187	2.269	1.411	1.443

*** $p < 0.05$**
