# Supplementary material for: Heat Exposure, Heat-Related Symptoms and Coping Strategies among Elderly Residents of Urban Slums and Rural Vilages in West Bengal, India
Source: Int J Environ Res Public Health. 2022 Sep 29;19(19):12446. doi: 10.3390/ijerph191912446 (PMC9564637; doi:10.3390/ijerph191912446)
Supplement: Supplementary file 1 [file ijerph-19-12446-s001.zip › Supplemental File S9. Odds Ratios Relating characteristics to Coping Strategies.pdf]

Supplemental File S9. Odds ratios relating different participant characteristics to reported coping strategies

	Electric Fan	Rest	Drink Water	Move	Hand Fan	Alter Social Activity
Age (reference = 60 – 69 years)						
70 years & older	1.8196	1.0412	0.8073	**0.6416**	**0.6029**	**0.5859**
Location (reference = Kolkata slums)						
Rural Villages	**0.2404**	**1.8473**	**2.3066**	**23.6578**	1.0522	**34.0130**
Gender (reference = men)						
Women	1.4685	1.0057	0.9226	0.7033	0.8760	**0.5028**
Marital Status (reference = married)						
Not currently married	**2.7633**	0.6410	**0.3310**	**0.2476**	0.8220	**0.1162**
Education (reference = none)						
Primary only	0.9663	1.3830	1.1101	0.7123	0.7633	0.8058
Some secondary	1.1774	0.6079	0.8989	0.8013	**1.6653**	0.7974
completed secondary	0.4737	2.4468	1.7725	0.5342	1.8457	0.8506
post-secondary	**3.0375**	**0.3495**	0.8494	**0.4684**	0.7099	**0.4536**
Currently employed (reference = no)						
Yes	1.5072	0.6664	**0.4975**	**0.5926**	1.1827	0.8154
Tobacco use (reference category = nonuser)						
Current/former tobacco user	0.8036	0.8168	1.3425	0.9640	1.0694	1.3362
Activity (reference = inactive)						
Morning active	1.8312	0.9685	0.7143	1.3002	0.7031	**2.0312**
Afternoon active	0.8152	0.6282	0.6282	0.6194	0.5835	0.6782
Active all day	0.8329	0.8961	0.8961	**2.4308**	**0.5345**	**1.9679**

	Remove/ Change Clothing	Add Food Item	Avoid or Reduce Activities	Take Shower or Bath	Delete Food Item
Age (reference = 60 – 69 years)					
70 years & older	1.1298	**0.5478**	**0.6366**	0.9927	**0.6243**
Location (reference = Kolkata slums)					
Rural Villages	**2.2728**	**0.2290**	**52.0800**	**32.6333**	**3.8333**
Gender (reference = men)					
Women	**0.3788**	**1.9838**	**0.5336**	**0.5593**	0.8970
Marital Status (reference = married)					
Not currently married	**0.5008**	**2.3289**	**0.0756**	**0.0584**	**0.3554**
Education (reference = none)					
Primary only	1.4281	**2.7625**	0.8676	1.1391	1.3030
Some secondary	1.0154	**1.8338**	1.2012	1.4330	**1.9254**
completed secondary	2.0778	1.9860	0.8045	**3.5000**	**2.8667**
post-secondary	1.6133	1.6078	1.0429	1.1587	1.8696
Currently employed (reference = no)					
Yes	0.9340	**0.5924**	0.7402	0.8035	0.9065
Tobacco use (reference category = nonuser)					
Current/former tobacco user	1.2588	0.7012	1.1282	0.9221	0.6274
Activity (reference = inactive)					
Morning active	0.6148	**0.3218**	**1.9105**	**1.8178**	1.2513
Afternoon active	**0.3708**	0.7483	0.5960	0.8375	1.1854
Active all day	0.7963	**0.3682**	**2.0014**	**2.7759**	1.1404

Statistically significant odds ratios ($p < 005$) indicated in bold
